# Eicosapentaenoic acid/arachidonic acid ratio and smoking status in elderly patients with type 2 diabetes mellitus

**DOI:** 10.1186/1758-5996-6-85

**Published:** 2014-08-13

**Authors:** Kenta Okada, Kazuhiko Kotani, Hiroaki Yagyu, Shun Ishibashi

**Affiliations:** Division of Endocrinology and Metabolism, Department of Internal Medicine, Jichi Medical University, 3311-1 Yakushiji, Shimotsuke-City, Tochigi, 329-0498 Japan; Department of Public Health, Jichi Medical University, 3311-1 Yakushiji, Shimotsuke-City, Tochigi, 329-0498 Japan; Department of Internal Medicine, University of Tsukuba Institute of Clinical Medicine, 3-2-7 Miya-machi, Mito, Ibaraki, 310-0015 Japan

**Keywords:** Arachidonic acid, Docosahexaenoic acid, Eicosapentaenoic acid, Older people, Smoking

## Abstract

**Background:**

A low ratio of eicosapentaenoic acid (EPA)/arachidonic acid (AA) is considered a risk factor for cardiovascular disease. Smoking is also a risk factor for cardiovascular disease even in an elderly population. This study investigated the relationship between EPA/AA ratio and smoking status among elderly patients with type 2 diabetes mellitus (T2DM).

**Findings:**

A total of 188 elderly patients with T2DM (men/women, 114/74; mean age, 65.0 ± 7.5 years) were studied in terms of their smoking status, diabetic conditions, and blood data, including EPA and AA. Current smokers showed a lower EPA/AA ratio than non-smokers (current smokers: 0.29, n = 49; non-smokers: 0.39, n = 139, *p* < 0.01). This relationship remained significant after adjusting for multiple variables.

**Conclusions:**

Smoking may affect the EPA/AA ratio among elderly patients with T2DM, suggesting a possible mechanism of cardiovascular disease development and indicating the importance of smoking secession in such patients.

## Introduction

Cardiovascular disease (CVD) is a crucial pathology and target for preventative action in patients with diabetes mellitus (DM) [1]. The eicosapentaenoic acid (EPA)/arachidonic acid (AA) ratio in blood is considered a promising marker of myocardial infarction and cardiac death [2–4] with a high EPA/AA ratio predicting a low CVD risk [2, 3].

Smoking is a major risk factor for CVD in DM [5–8], the adverse effects of which on the cardiovascular system can accumulate in elderly patients. Thus, in preventing CVD events, the cessation of smoking in patients aged over 50 years is considered to be equally as important as in younger patients [9]. The association between EPA/AA ratio and smoking status, especially in elderly patients with type 2 DM (T2DM) has yet to be investigated, and such information would be useful in clinical practice. The present study aimed to investigate such an association in elderly patients with T2DM [9].

## Methods

A total of 188 patients aged over 50 years, diagnosed with T2DM (men/women, 114/74; mean age, 65.0 ± 7.5 years) and not taking medications containing EPA and AA were examined. Patients were excluded who had a history of CVD event (s), a recent acute illness, systemic inflammatory disease, severe nephropathy (i.e., stage 3 to 5), liver dysfunction, or type 1 DM. The study was approved by the Jichi Medical University Ethical Committee.

Hypertension was determined as systolic blood pressure of ≥ 140 mmHg, diastolic blood pressure ≥90 mmHg, and/or anti-hypertensive drug use [10]. Nephropathy was defined by a urinary albumin-to-creatinine ratio of ≥ 30 mg/g creatinine [11]. Fasting blood samples were collected at our outpatient clinic in order to measure the levels of the following parameters: glucose, hemoglobin A1c (HbA1c), total cholesterol, triglyceride, high-density lipoprotein (HDL) cholesterol, EPA, docosahexaenoic acid (DHA), and AA.

High-performance liquid chromatography (HLA-723G8; Tosoh, Tokyo, Japan) was used to measure HbA1c. Serum samples were collected in a heparinized poly-tube, lipids were extracted by Folch’s procedure, and fatty acids (tricosanoic acid (C23:0) was used as an internal standard) were methylated with boron trifluoride and methanol. EPA, DHA, and AA levels in methylated fatty acids were analyzed by gas chromatography (GC-2010; Shimadzu, Kyoto, Japan) with a capillary column (TC-70; GL Sciences Inc., Tokyo, Japan). Smoking habits were confirmed via self-reporting.

Differences between current smokers and non-smokers were analyzed using the t-test and Chi-square test. A multivariable-adjusted analysis on the association between EPA/AA ratio and smoking status was performed using a general linear model (SPSS software, SPSS Inc., IL, USA). Parameters with skewed distributions were log-transformed in all analyses. A *p*-value of < 0.05 was considered to be significant.

## Results

Current smokers were significantly younger in age, with a greater proportion of males than non-smokers (Table 1). They also exhibited a higher percentage of hypertension and neuropathy, a higher level of HbA1c, and a lower HDL cholesterol level than non-smokers. There were no significant differences in AA, DHA, and EPA levels between current smokers and non-smokers.

**Table 1:** **Clinical characteristics**

Parameters	All (n = 188)	Non-smokers (n = 139)	Current smokers (n = 49)	***P***
Age, years	65.0 ± 7.5	65.9 ± 7.4	62.4 ± 7.3	<0.01**
Gender, men/women	114/74	71/68	43/6	<0.01**
Body mass index, kg/m^2^	25.5 ± 4.3	25.6 ± 4.4	25.2 ± 3.8	0.59
Hypertension, n (%)	134 (71%)	93 (67%)	41 (84%)	0.03*
Glucose, mg/dL	137 ± 47	138 ± 49	135 ± 40	0.64
Hemoglobin A1c, %	7.3 ± 0.9	7.2 ± 0.9	7.6 ± 1.0	0.03*
Insulin therapy, n (%)	55 (29%)	41 (29%)	14 (29%)	0.90
Retinopathy, n (%)	78 (41%)	56 (40%)	22 (45%)	0.57
Neuropathy, n (%)	107 (57%)	73 (53%)	34 (69%)	0.04*
Nephropathy, n (%)	76 (40%)	53 (38%)	23 (47%)	0.28
LDL-cholesterol, mg/dL	92 ± 28	92 ± 27	91 ± 33	0.87
HDL-cholesterol, mg/dL	61 ± 16	63 ± 16	56 ± 16	<0.01**
Triglycerides, mg/dL	107 (73-148)	101 (67-148)	121 (84-153)	0.05
Statin therapy, n (%)	83 (44%)	63 (45%)	20 (41%)	0.59
AA, μg/mL	173 (143-210)	170 (141-207)	180 (150-232)	0.31
DHA, μg/mL	128 (109-176)	129 (111-176)	128 (98-177)	0.15
DHA/AA ratio	0.78 (0.60-1.04)	0.81 (0.62-1.07)	0.71 (0.55-1.00)	0.06
EPA, μg/mL	61 (42-101)	62 (43-109)	56 (34-92)	0.06
EPA/AA ratio	0.37 (0.23-0.63)	0.39 (0.24-0.65)	0.29 (0.18-0.46)	<0.01**

*LDL*: low-density lipoprotein, *HDL*: high-density lipoprotein, *AA*: arachidonic acid, *EPA*: eicosapentaenoic acid, *DHA*: docosahexaenoic acid.

Data are the means ± standard deviations, medians (interquartile ranges) or numbers (%).

*p < 0.05, ** *p* < 0.01: comparison between current smokers and non-smokers (t-test or Chi-square test).

Of note, a significantly lower EPA/AA ratio was demonstrated in current smokers than in non-smokers (median, 0.29 versus 0.39, *p* < 0.01). Weak correlations between EPA/AA ratio and other variables (age, gender, BMI, hypertension, HbA1c, insulin therapy, complications, lipids, and statin therapy) were observed for the entire population (*p* > 0.05 in all, data not shown). A significant difference in the EPA/AA ratio between groups (*p* = 0.02) remained after adjusting for multiple variables (age, gender, BMI, hypertension, HbA1c, insulin therapy, complications, lipids, and statin therapy).

## Discussion

This study found current smokers to have a lower EPA/AA ratio than non-smokers among elderly patients with T2DM, indicating that smoking can detrimentally affect the EPA/AA balance in such patients. Considering that the EPA/AA ratio is a predictor of CVD events in patients with DM [2, 3] and smoking cessation is an achievable and effective option for preventing CVD [7, 8], the findings in this study strongly support the importance of smoking cessation in the management of CVD among elderly patients with T2DM.

Smoking results in various oxidants that are capable of producing free radicals and lipid peroxidation [12]. Patients with T2DM are especially known to have high levels of these oxidants [13]. Polyunsaturated fatty acids, particularly omega-3 acid ethyl esters, undergo oxidation, leading to a reduction in EPA in the presence of oxidants [14–16]. A previous report has indicated that AA levels in smokers (mean age, 50 years) are decreased [17], which is inconsistent with the findings of our study; however, another report indicated that AA in smokers are not decreased [18]. Of note, the metabolism of AA is not always parallel to that of EPA [19–21], and compared to EPA, smoking has been shown to delay the conversion rate of AA to eicosanoids [22]. These findings may support an impairment of EPA/AA balance, namely, a decrease in the EPA/AA ratio, observed among smokers in our study. Our results should be further analyzed using additional markers, such as oxidative stress markers, in order to clarify the underlying mechanisms.

Similar to EPA, DHA is also an omega-3 polyunsaturated fatty acid. The pathological mechanism underlying the difference between EPA and DHA is unclear. However, clinical studies have reported that lower levels of EPA, but not DHA, were significantly associated with all-cause mortality [23], and that there was no clear association between the DHA/AA ratio and cardiovascular risk [24]. The results of our study may be in line with these studies [23, 24].

Some reports have shown that smoking worsens blood pressure [25], glycemic control [26], and neuropathic conditions [27, 28]. Similar findings were observed in our study. However, from the results of our adjusted analyses, these variables do not seem to have a major influence on the association between smoking status and EPA/AA ratio.

This study has certain limitations. This was a cross-sectional study; thus, further intervention studies are required. Although we saw a significant difference in the association between smoking status and EPA/AA ratio, the small patient number might present a problem in terms of a lower statistical power; thus, a larger-scale study is required. Additionally, as there is no information regarding dietary fish consumption and the dose and duration of smoking (e.g., the Brinkman index), and type of cigarette were not investigated in detail, these must be included into future work.

In summary, the present study suggests that current smoking status and a low EPA/AA ratio may enhance CVD risk in elderly patients with T2DM. Considering an individual’s smoking status coupled with EPA/AA ratio may be important in the management of T2DM, especially in the elderly. Further studies are expected.

## Authors’ information

Kazuhiko Kotani, Hiroaki Yagyu, Shun Ishibashi are co-authors.

## Abbreviations

CVD: Cardiovascular disease
DM: Diabetes mellitus
T2DM: Type 2 diabetes mellitus
EPA: Eicosapentaenoic acid
AA: Arachidonic acid
DHA: Docosahexaenoic acid
HbA1c: Hemoglobin A1c
HDL: High-density lipoprotein.
